# The effect of agility exercise on eicosanoid excretion, oxidant status, and plasma lactate in dogs

**DOI:** 10.1186/1746-6148-8-249

**Published:** 2012-12-28

**Authors:** Wendy I Baltzer, Anna M Firshman, Bernadette Stang, Jennifer J Warnock, Elena Gorman, Erica C McKenzie

**Affiliations:** 1 Departments of Clinical Sciences (Baltzer, Firshman, Stang, Warnock, McKenzie) and Biomedical Sciences (Gorman), College of Veterinary Medicine, Oregon State University, Corvallis, OR, 97331, USA; 2 Present address: 225 Veterinary Medical Center, 1365 Gortner Ave, St. Paul, MN, 55108, USA

**Keywords:** Agility exercise, Dog, Urinary isoprostane, Plasma lactate, Oxidant stress, Urinary eicosanoids, Thromboxane B_2_

## Abstract

**Background:**

The objective was to determine the effects of agility exercise on dogs of different skill levels with respect to urinary eicosanoids, urinary 15F_2t_-isoprostane (lipid peroxidation marker) and hematological/biochemical changes in plasma. Fifteen adult dogs had blood and urine samples obtained prior to, immediately and 4-hours following an agility exercise.

**Results:**

Hematocrit, red blood cells (RBC), albumin, and hemoglobin increased following exercise, with greatest increases correlating to increased skill group (novice, intermediate, masters); at 4-hours post-exercise, hematocrit, RBC, and hemoglobin were decreased. Phosphorus increased following exercise with the greatest increase in novice and intermediates. Plasma lactate increased 3.6-fold in masters, 3.2-fold in intermediates, and 1.2-fold in novice dogs. Urine thromboxane B_2_ (TXB2) more than tripled 4-hours post-exercise while 6-keto prostaglandin F_1α_ (PGF1α, prostacyclin metabolite), prostaglandin E_2_ metabolites (13,14-dihydro-15-keto-prostaglandin A_2_ and 13,14-dihydro-15-keto-prostaglandin E_2_), and 13,14-dihydro-15-keto prostaglandin F_2α_ were unaffected as determined by a competitive enzyme immunoassay and standardized by division with urine creatinine. Urine 15F_2t_-isoprostane increased insignificantly.

**Conclusions:**

Alterations in the plasma post-exercise were likely due to hemoconcentration from insensible water loss, splenic contraction and sympathetic stimulation while 4-hours later autohemodilution reduced RBC parameters. Elevations in plasma lactate and urinary TXB2 correlated with advanced skill level/speed of the dogs.

## Background

The United States Dog Agility Association (USDAA) alone, registers over 25,000 competitors and organizes over 400 days of agility competitions each year in the United States, Puerto Rico, Canada, Mexico, Bermuda, and Japan [1]. The percentage of dogs injured during agility exercise is estimated at 33% [2]. With such a high rate of injury, determining the effects of this type of exercise on the physiologic responses of dogs including the degree of oxidant stress and eicosanoid production induced by this exercise, is important to understanding why injury rates are so high.

Canine agility exercise has been shown to induce significant increases in blood lactate and hence an anaerobic metabolic state [3,4]. In addition, plasma chloride, hematocrit, and triglycerides increase with agility exercise whereas, serum albumin, calcium and phosphorus concentrations decrease immediately after this exercise [3,4]. No alteration in plasma protein, sodium, potassium or creatinine concentrations has been found with agility exercise in dogs [4]. The alterations that do occur have been attributed to splenic contraction in addition to increased movement of fluid from the interstitium into the vascular space, and subsequently into contracting muscle cells [4]. Therefore, there is an overall minimal change in plasma volume in dogs during agility exercise but a tremendous necessity for anaerobic metabolism. A wide variety of dog breeds participate in agility events, and there are a wide variety of competition levels in the sport. Hence, due to differences in fitness and innate athletic ability, some dogs may experience greater effects of exertion than others during physical activity in agility. The effect of skill and intensity level on specific physiologic variables in agility competition dogs is unknown.

Exercise increases the rate of oxygen consumption, subsequently resulting in the production of free radicals or reactive oxygen species. The reactive oxygen species cause lipid peroxidation and cell injury, which may overwhelm cellular antioxidant mechanisms [5]. In humans, intense exercise induces acute oxidant stress, reflected by increased 15F_2t_ isoprostane excretion in urine [6-9]. Isosprostanes form when arachidonic acid reacts with reactive oxygen species in cell membranes and they are considered a marker of oxidant activity and lipid peroxidation *in vivo* in humans and several other species [10]. The period of time required for recovery from exercise-induced oxidant damage is unknown, but in humans, isoprostane production returned to baseline within 24 hours following exercise [11,12]. In sled dogs undergoing endurance exercise, plasma isoprostanes increase following exercise and correlate with the logarithm of serum creatinine kinase activity, a marker of muscle damage [13]. To the authors’ knowledge, the excretion of 15F_2t_ isoprostanes in the urine of dogs following agility exercise has not been previously reported nor have hematological and biochemical parameters been followed for 4-hours post agility exercise and compared between skill groups.

Exercise in horses and humans induces an inflammatory response and has been shown to alter eicosanoid production [14,15]. Dogs experience an anaerobic metabolic state during agility exercise with increases in plasma lactate, hemoglobin, hematocrit, and red blood cell count, however to the authors’ knowledge, the effects of agility exercise on eicosanoid production in dogs of different skill levels has not been previously investigated [3].

The purpose of this study was to determine the effects of agility exercise on dogs of different skill levels with respect to 15F_2t_ isoprostane, thromboxane B_2_ (TXB2), 6-keto prostaglandin F_1α_ (PGF1α, prostacyclin metabolite), prostaglandin E_2_ metabolites (13,14-dihydro-15-keto-prostaglandin A_2_ and 13,14-dihydro-15-keto-prostaglandin E_2_), and 13,14-dihydro-15-keto prostaglandin F_2α_ excretion in the urine and systemic hematological and biochemical changes in the plasma; and to determine if alterations in these parameters had resolved within 4 hours of the high intensity exercise.

## Methods

### Animals

The study protocol was approved by the Oregon State University Animal Care and Use Committee and written informed consent was obtained from all owners of dogs participating in the study prior to participation of the dogs. A local agility dog club was contacted and volunteers were requested to participate in the study. Dogs were reported by their owners to be competitors in agility either at the novice (referred to as starters by the USDAA), intermediate (advanced), or masters classes. Dogs earn entry into each class through competition and their scores in those competitions [1]. Owners reported how often their dogs trained on a weekly basis and if their dogs were currently receiving any medications. The sex, age and breed of each dog were also recorded. Each dog was examined by a veterinarian prior to participating in the agility exercise during which the heart and lungs were auscultated and their gait was examined for signs of lameness or dysfunction. Dogs were excluded from the study if they were receiving electrolyte supplements, exhibited signs of lameness the day of the experiment, or had an elevated body temperature prior to beginning the experiment (>103.0°F, 39.4°C). Dogs were not allowed to eat within 3 hours prior to the agility test but they were allowed training treats in small amounts following the test. A full meal was not given to any dog until after the 4-hour sample collection was performed. At no time was water consumption restricted from the dogs during the assessment.

### Agility exercise test

Prior to the agility test, a heart rate monitor (S625x, Polar Electro Inc, Lake Success, NY) was placed around the girth of each dog using an elastic harness. Each dog ran a course, consisting of 11 jumps and 4 tunnels, 3 times with a 5 minute rests period between the first and second, then second and third runs. The dogs ran the course three times to duplicate the exercise they would experience during an agility competition. The height of the jumps was adjusted for each dog with dogs less than 12 inches in height at the shoulder jumping 12 inch tall jumps, dogs 12 to 16 inches tall jumping 16 inch tall jumps, dogs 16 to 21 inches tall jumping 22 inch tall jumps and dogs over 21 inches jumping 26 inch tall jumps [1]. Each dog ran the course 3 times in succession with a 5-minute rest period between runs one and two and two and three. Each dog was allowed to sit, lie down, or walk during the rest period, but not trot or run. Each run was timed in seconds with a stopwatch and the times recorded for each dog. The number of missed jumps or obstacles taken out of sequence was also recorded for each dog. Heart rate, respiratory rate, and rectal temperature were recorded prior to, immediately following and 4 hours following the 3 runs through the agility course.

### Sample collection

Blood was collected from each dog within 2 hours prior to participating in the agility exercise, and within 2 minutes of completing the agility exercise test, and 4-hours after completing the test via jugular venipuncture. Blood was placed into EDTA and lithium heparin tubes immediately following collection, and heparinized blood was immediately submitted for determination of venous blood lactate using an automated blood gas analyzer (RapidLab 1265, Siemens Healthcare Diagnostics, Tarry Town, NY) for determination of venous blood lactate. Packed cell volume and total protein were determined immediately following collection. Hematological analyses were performed using the ADVIA 120 hematology analyzer (Siemens, Deerfield, IL), and biochemical parameters with the Hitachi 917 chemistry analyzer (Roche Group, Tucson. AZ). All hematological and biochemical analyses were performed within 4 hours of sample collection. Urine was collected from each dog by free catch technique within 2 hours prior to the agility exercise test, within 1 hour following the test, and within one-half hour of 4 hours following the exercise test. Urine was stored within 1 hour of collection at -80°C until analysis.

### Isoprostane determination

Samples were analyzed in duplicate and in batches according to the manufacturer’s instructions to reduce interassay variation. The isoprostane assay and urinary creatinine assays have undergone extensive analytic validation by the manufacturers, as indicated in the results provided in the package insert. Urine 15F_2t_ isoprostane (ISO) was determined using a competitive enzyme immunoassay (15F_2t_ isoprostane enzyme immunoassay kit, Cayman Chemical Co, Ann Arbor, Mich) after sample pre-treatment with β-glucuronidase (Oxford Biochemical Research, Oxford, MI). A spectrophotometric plate reader was used at 420nm. The specificity of the EIA is reported by the manufacturer as 100% cross reactivity for 8-isoprostane and 8-iso prostaglandin F_2α_ ethanolamide, 20.6% for 8-iso prostaglandin F_3α_, and 4% for 2,3-dinor-8-iso porstaglandin F_2α_. Samples were analyzed in duplicate and in batches to reduce interassay variation. This assay has undergone extensive analytic validation by the manufacturers, as indicated in results provided in the package insert. Intra-assay coefficient of variation ranged from 19.9: at 2 pg/ml to 12.6% at 500 pg/ml and inter-assay coefficient of variation ranged from 9.6% at 2 pg/ml dose to 10.5% at 500 pg/ml. In-house validation was performed with urine samples previously collected from healthy dogs as part of unrelated, previously published research in dogs [16]. Our previous validation of the assay included the assessment of dilutional parallelism of canine urine samples (5 samples at 3 dilutions in which we determined the correct dilution for samples such that they would be within the working range of the assay) and determination of interassay variability (5 samples assayed on 5 separate days) [16].

### Urinary eicosanoid determination

Urine samples were kept at -20°C until analysis for their prostanoid content. Thromboxane B_2_ (TXB2), 11-dehydro-thromboxane B_2_ (11-TXB2), 6-keto prostaglandin F_1α_ (PGF1α, prostacyclin metabolite), prostaglandin E_2_ metabolites (13,14-dihydro-15-keto-prostaglandin A_2_ and 13,14-dihydro-15-keto-prostaglandin E_2_), and 13,14-dihydro-15-keto prostaglandin F_2α_ (PGF2α) are not species-specific compounds and urinary excretion was determined using ACE™ competitive enzyme immunoassay (Cayman Chemical, Ann Arbor, MI) per the manufacturer’s instructions. Samples were analyzed in duplicate and batched to reduce interassay variation. The TXB2 and 11-TXB2 assays have undergone extensive analytic validation by the manufacturers and use of 11-TXB2 in canine urine has been validated previously [17]. The PGF1α assay for prostacyclin excretion has undergone extensive analytic validation by the manufacturers and with cross reactivity to prostaglandin D2 <0.01%, prostaglandin F2α=11%, and TXB2 =0.05%. The prostaglandin E2 metabolite assay used is recommended for analysis of urine for metabolites of PGE2 since PGE2 is rapidly converted in vivo to its 13,14-dihydro-15-keto metabolite which is unstable and rapidly degraded to 13,14-dihydro-15-keto PGA_2_. The assay used to determine 13,14-dihydro-15-keto-prostaglandin A_2_ and 13,14-dihydro-15-keto-prostaglandin E_2_ concentrations in the urine and has been validated by the manufacturer with cross-reactivity to prostaglandin D2, prostaglandin F2α, 6-keto-prostaglandin F1α, prostaglandin F1α, and TXB2 <0.01%. The PGF2α assay has been used for direct quantitation of 13,14-dihydro-15-keto prostaglandin F_2α_ in urine and the manufacturer has performed extensive analytical validation of the assay with cross reactivity for prostaglandin D2, 6-keto-prostaglandin F1α, prostaglandin F1α, and TXB2<0.01%.

Urine creatinine concentration was determined using DetectX™ competitive enzyme immunoassay (Arbor Assays LLC, Ann Arbor, MI) with plates read at spectrophotometer settings at 490nm as per the manufacturer’s instructions. This assay has been validated for use with dog urine by the manufacturer and the intra-assay and inter-assay coefficients of variation were reported as 3.0% and 3.9% or less, respectively. Results for ISO are reported as pg/mL divided by creatinine in mg/dL. Results for TXB2, PGF1α, prostaglandin E2 metabolites, and PGF2α are reported as pg/mL divided by creatinine in mg/dL.

### Statistical analyses

Values are reported as the mean ± standard deviation and median, range. A Nemenyi-Damico-Wolfe-Dunn Test was used to detect differences between sample times (baseline prior to exercise, immediately post-exercise, and 4-hours post-exercise) and skill levels of the dogs (novice, intermediate, masters) [18]. Significance was set at P < 0.05.

## Results

### Skill groups

Fifteen dogs were enrolled in the study. Masters dogs were an average of 6.2 ± 2.0 (median 7.0, range 4-8) years of age (n= 5). Novice dogs were 2.8 ± 1.5, median 2.5, range 1-5 years, n= 6) which was slightly younger than intermediate level dogs (7.0 ± 2.3 years, median 7.0 and range 5-9, n= 4; P>0.05). Seven of the 15 dogs were spayed females and the rest were neutered males. The masters level dogs had spent 4.0 ± 1.4 years performing agility (median 5.0 and range 2-5 years), the intermediate level dogs 3.0 ± 0.8 (median 3.0, range 2-4) years and the novice level dogs 1.2 ± 1.1 (median 0.8, range 0.2-3) years. The height of the jumps was not different between the skill groups (novice 16 ± 4.4 (16, 12-20) inches, intermediate 16 ± 0.0 (all dogs 16 inches), masters 15.2 ± 5.2 (16, 8-20) inches, P>0.05). The breeds in the masters level group included two mixed breeds, a Border Collie, a Jack Russell Terrier, and a Pembroke Welsh Corgi. The intermediate level dogs contained a Golden Retriever, a Belgian Terrier, an Australian Shepherd, and a Standard Poodle. The novice level group contained a Golden Retriever, a Boxer, a Standard Poodle, a Miniature Poodle, a Welsh Terrier, and a Queensland Heeler. Six of the 15 dogs were administered glucosamine and chondroitin sulfate supplements daily. Two dogs were intermittently treated with antibiotics and antihistamines for owner reported skin allergies. Two other dogs had been previously diagnosed as hypothyroid and were receiving thyroid supplementation that upon testing resulting in serum T4 concentrations within the reference range for the lab. The frequency with which each group trained their dogs was not different between the groups and on average the masters level dogs spent 4.2 ± 1.8 (median 4.0, range 2-6) hours per week in training while the intermediate level dogs spent 4.0 ± 1.4 (3.5, 3-6) hours and the novice level dogs 3.0 ± 0.9 (3.0, 2-4) hours per week in training, respectively, P>0.01.

The time taken to complete the course was significantly different between the skill groups (two-way ANOVA, P = 0.0092) and for each run (run 1, run 2 or run3, P = 0.01). The masters level dogs took the least time to complete their runs [run 1, 29.5 ± 9.5 (median 25.4, range 23.8-46.3), run 2, 29.4 ± 10.0 (25.3, 22.9-44.2), run 3, 29.2 ± 8.4 (25.8, 23.6-43.8) seconds] and had the least mistakes (such as missed jumps) compared to the intermediate level [run 1, 38.8 ± 9.7 (median 39.8, range 27.9-47.8), run 2, 41.3 ± 18.2 (32.9, 30.8-68.4), run 3, 49.8 ± 39.5 (30.4, 29.4-109) seconds] or novice level dogs (run 1 33.6 ± 7.5 (31.5, 26.2-45.0), run 2, 33.2 ± 4.8 (32.4, 28.2-40.5), run 3, 34.3 ± 1.0 (34.4, 30.7-37.5) seconds).

### Physical variables

Body temperature increased significantly immediately following the exercise test, but returned to pre-exercise values by 4-hours after the test (pre-exercise 101.4 ± 0.8, median 101.3, range 100.2-102.7°F, immediately post-exercise 102.4 ± 1.3, median 102.1, 100.3-103.6°F, and 4-hours post 100.4 ± 1.0, median 100.8, range 98.1-101.7°F, P<0.0001). Body temperature was unaffected by skill level (P=0.3). Heart rate was significantly higher immediately following the exercise test and decreased significantly 4 hours after the test compared to pre-test and immediate post-test values for all groups of dogs (pre-exercise 104 ± 21.5, median 96, range 84-148, immediately post-exercise 136.5 ± 19.7, median 130, range 110-180, 4-hours post 83.6 ± 25.9, median 74, range 52-133 beats per minute, P<0.0001). As with body temperature, heart rate was not affected by the dogs’ skill level (P=0.07).

### Hematological profile

Results of the hematology analysis are reported in Table 1. Red blood cell count (RBC), hematocrit (HCT), hemoglobin, and serum albumin increased immediately following the agility exercise (P<0.05). Albumin decreased to values similar to pre-exercise values by 4-hours following the exercise test, however, RBC, HCT and hemoglobin were decreased at 4-hours compared to pre-exercise values. RBC, HCT, hemoglobin, and albumin were different in different skill groups; they increased the greatest in the masters group followed by intermediates and the least in the novice dogs immediately after exercise (P<0.05). None of the other hematological parameters examined (white blood cell count, total plasma protein, platelet count, mean corpuscular volume, mean corpuscular hemoglobin concentration, neutrophil count, lymphocyte count, monocyte count, eosinophil count, basophil count) were affected by time or skill level of the dogs, P>0.05.

**Table 1:** Hematological profile results

		**Novice**			**Intermediate**			**Masters**		**Reference range**
	**Pre**	**Post**	**4 hr Post**	**Pre**	**Post**	**4 hr Post**	**Pre**	**Post**	**4 hr Post**	
**RBC § (x10**^**6**^**/μL)**	7.0 ± 0.6;	7.3 ± 0.2;	6.8 ± 0.6;	6.3 ± 0.9;	7.0 ± 1.0;	6.1 ± 0.7;	6.2 ± 0.3;	7.1 ± 0.4;	6.1 ± 0.4;	5.5-8.5
	7.1, 6-7.6	7.3, 6.9-7.5	6.7, 6.2-7.6	6.6, 5-6.9	6.9, 5.8-8.2	6.2, 5.1-6.8	6.4, 5.8-6.5	6.9, 6.6-7.7	6.2, 5.6-6.5	
**HCT (%)§**	47.3 ± 3.7;	49.1 ± 1.3;	45.3 ± 2.8;	42.1 ± 5.4;	47.4±6.2;	40.9 ± 5.0;	43.5 ± 3.1;	49.7 ± 4.0;	42.7 ± 3.1;	37-55
	48.1, 41.4-52.1	47-50.9	45.3, 42-49.5	43.9, 34.2-46.3	46.4, 41-55.8	41.4, 34.5-46.3	44.3, 38.7-47.1	50.2, 43.8-54.4	38.7-46.6	
**Hgb (g/dL) §**	16.9 ± 1.3;	17.7 ± 0.3;	16.4 ± 1.1;	15.4 ± 1.6;	16.8 ± 2.4;	14.7 ± 1.8;	15.9 ± 0.9;	18 ± 1.2;	15.4 ± 1.0;	12-18
	17.2, 14.7-18.4	17.7, 17.2-18.1	16.3, 15.3-17.3	15.8, 13-16.8	16.4, 14.4-20	14.8, 12.4-16.7	16, 14.5-16.8	17.9, 16.7-19.8	15.3, 14.1-16.7	
**WBC (x103/μL)**	8.9 ± 2.9;	9.4 ± 3.2;	9.6 ± 3.1;	6.7 ± 1.5;	8.2 ± 1.3;	7.2 ± 1.4;	6.5 ± 1.3;	7.5 ± 1.8;	7.1 ± 1.5;	6-17
	8.0, 6.5-14.1	8.3, 6.1-15	9.2, 6.7-15.2	6.7, 5.1-8.3	8.7, 6.3-9.2	7.3, 5.5-8.5	6.2, 5.2-8.5	6.4, 6.2-10.4	7.5, 5.1-8.6	
**Platelets (x1000/μL)**	269 ± 76;	263 ± 87;	236 ± 68;	253 ± 27;	274 ± 41;	236 ± 31;	286 ± 46;	319 ± 58;	242 ± 85;	200-900
	272, 141-347	267, 127-354	241, 156-314	251, 226-284	272, 229-324	238, 200-269	293, 211-334	233-391	279, 115-333	
**Albumin § (g/dL)**	3.8 ± 0.2;	3.9 ± 0.1;	3.9 ± 0.2;	3.7 ± 0.1;	3.9 ± 0.1;	3.7 ± 0.1;	3.6 ± 0.1;	3.8 ± 0.1;	3.6 ± 0.1;	2.3-4.0
	3.8, 3.6-4.0	3.9, 3.7-4.1	3.8, 3.6-4.1	3.7, 3.6-3.8	3.9, 3.8-4.1	3.7, 3.6-3.9	3.6, 3.6-3.8	3.8, 3.6-4.0	3.7, 3.5-3.8	
**Total Plasma Protein (g/dL)**	6.1 ± 0.4	6.2 ± 0.3;	6.1 ± 0.5;	6.5 ± 0.5 6.5	6.5 ± 0.4;	6.1 ± 0.4;	6.2 ± 0.3	6.5 ± 0.2;	6.0 ± 0.3;	5.4-7.6
	6.1, 5.6-6.8	6.2, 5.8-6.6	6.0, 5.5-6.8	5.8-7.0	6.6, 6.1-6.8	6.1, 5.6-6.5	6.0, 6.0-6.6	6.5, 6.1=6.7	6.1, 5.7-6.4	

Values are mean ± standard deviation, median and range; differences were detected using the Memenyi-Damico-Wolfe-Dunn Test **§** Parameter significantly affected by the exercise test (i.e. pre or post exercise test, P<0.05); ¶ parameter significantly different between skill groups, P<0.05. Pre = pre-exercise, Post = post-exercise, 4 hr post = 4 hours following exercise, RBC= red blood cell count, HCT= hematocrit, Hgb= hemoglobin, WBC= white blood cell count, μL= microliter, g/dL= grams per deciliter.

### Biochemistry profile

Biochemistry parameters are reported for each skill group and time point in Table 2. Serum creatinine and total plasma protein were not affected by the exercise test or skill level (P>0.05). Lactate increased significantly immediately after the exercise test (P<0.001) and differed between skill groups, Table 2 (P<0.001). Lactate increased most in the masters skill group, then the intermediate group, and it changed little in the novice dogs following agility exercise. Phosphorus increased in dogs immediately after the exercise test and continued to rise 4-hours following the test, with the greatest increase in novice and intermediate dogs and least change in the masters group (P<0.05). Creatinine kinase activity increased slightly but not significantly following exercise in all groups (pre-exercise 124.0 ± 12.9, immediately post-exercise 139.1 ± 22.2, 4 hours post-exercise 190.8 ± 37.7 U/L). Creatinine kinase was affected by skill level of the dogs with the novice group having the highest values (P<0.05, Table 2). Blood urea nitrogen, calcium, sodium, potassium and chloride did not significantly change over time or between dog skill levels, P>0.05.

**Table 2:** Serum biochemistry profile results

		**Novice**			**Intermediate**			**Masters**		**Reference range**
	**Pre**	**Post**	**4 hr Post**	**Pre**	**Post**	**4 hr Post**	**Pre**	**Post**	**4 hr Post**	
**Lactate § (mmol/L)**	1.04 ± 0.35; 1.1, 0.58-1.47	1.28 ± 0.55; 1.22, 0.82-2.34	0.64 ± 0.20; 0.6, 0.40-0.97	2.34 ± 1.66; 1.6, 1.4-4.82	7.39 ± 5.43; 6.78, 1.42-14.60	2.10 ± 1.97; 1.55, 0.42-4.86	1.10 ± 0.41; 1.0, 0.64-1.69	3.93 ± 2.53; 3.56, 0.87-7.89	0.69 ± 0.27; 0.52, 0.49-1.05	< 4
**Creatinine (mg/dL)**	1.0 ± 0.3; 1.0, 0.7-1.3	0.9 ± 0.3; 1.0, 0.6-1.2	0.8 ± 0.3; 0.9, 0.5-1.1	1.0 ± 0.3; 1.1, 0.5-1.2	1.1 ± 0.4; 1.2, .5-1.4	0.9 ± 0.3; 0.9, 0.5-1.1	0.9 ± 0.4; 0.9, 0.6-1.5	1.0 ± 0.4; 1.0, 0.7-1.6	0.8 ± 0.3; 0.8, 0.7-1.4	1-2
**BUN (mg/dL)**	18. ±3; 19, 13-21	17 ± 3; 17, 12-21	16 ± 4; 16, 10-22	19 ± 4; 19, 14-24	19 ± 4; 20, 14-23	17 ± 4; 18, 12-21	17 ± 5; 17, 10-23	18 ± 5; 17, 12-23	18 ± 2; 18, 16-21	10-30
**Creatinine Kinase (IU/L)**	138 ± 59; 123, 88-248	100 ± 23; 103, 62-130	225 ± 158; 140, 102-448	110 ± 45; 98, 72-173	142 ± 105; 114, 63-281	150 ± 134; 91, 67-349	108 ± 52; 108, 48-189	136 ± 64; 131, 48-218	116 ± 49; 107, 50-180	50-300
**Calcium (mmol/L)**	10.5 ± 0.2; 10.6, 10.2-10.8	10.4 ± 0.4; 10.49.6-10.6	10.5 ± 0.1; 10.5, 10.4-10.7	10.3 ± 0.4; 10.4, 9.8-10.6	10.6 ± 0.2; 10.6, 10.3-10.8	10.2 ± 0.1; 10.2, 10.0-10.3	10.1 ± 0.1; 10.1, 9.9=10.3	10.3 ± 0.2; 10.3, 10.1-10.5	10.2 ± 0.4; 10.3, 9.6-10.7	8-12
**Phosphorus § (mmol/L)**	3.9 ± 0.5; 3.9, 3.3-4.7	4.2 ± 0.6; 4.3, 3.1-5	4.7 ± 0.2; 4.7, 4.4-4.9	3.3 ± 0.2; 3.3, 3.1-3.6	3.7 ± 0.3; 3.8, 3.3-4	4.2 ± 0.5; 4.2, 3.7-4.7	3.9 ± 0.7; 3.8, 2.9-4.8	3.9 ± 0.7; 3.7, 3.3-4	4.6 ± 0.6; 4.3, 4-5.4	3-7
**Sodium (mEq/L)**	146.8 ± 1.5 146.9, 144.4-149.2	148 ± 1; 149, 147-149	148 ± 3; 148, 146-154	146.7 ± 3.57 146.6, 142.5-151.2	151 ± 1; 151, 149-152	146 ± 5; 147, 140-150	145.2.6 ± 2.1; 146.1, 141.7-146.7	149 ± 1.6; 149, 146-150	147 ± 2; 148, 145-149	140-158
**Potassium (mEq/L)**	4.2 ± 0.2; 4.2, 3.9-4.3	4.4 ± 0.2; 4.5, 4-4.5	4.1 ± 0.8; 4.3, 2.5-4.8	4.1 ± 0.1; 4.1, 3.9-4.2	4.4 ± 0.5; 4.3, 4-5.1	4.1 ± 0.4; 4.2, 3.6-4.4	4.1 ± 0.2; 4.1, 3.9-4.4	4.3 ± 0.4; 4.3, 3.8-4.9	4.1 ± 0.4; 4.3, 3.5-4.4	4.0-5.7
**Chloride (mEq/L)**	114 ± 2; 114, 110-115	114 ± 2; 115, 111-116	113 ± 2; 113, 111-117	114 ± 5; 116; 106-118	113 ± 6; 115, 105-119	111 ± 6; 113, 103-117	114 ± 3; 114, 111-118	113 ± 2; 113, 110-114	114 ± 3; 115, 110-117	100-115
**ALT (IU/L)**	50 ± 31; 38, 27-111	50 ± 30; 40, 28-110	52 ± 33; 40, 29-119	41 ± 7; 42, 32-46	47 ± 9; 47, 36-57	45 ± 8; 47, 33-52	70 ±72; 31; 27-194	77.0 ± 76; 36, 31-209	73 ± 71; 37, 30-197	5-65
**ALP (IU/L)**	47 ±28; 35, 21-87	47 ± 29; 32, 22-89	43 ± 28; 29, 20-82	53 ± 32; 47, 32-46	58 ± 29; 55, 32-91	54 ± 30; 51, 27-87	30 ± 10; 28, 20-42	30 ± 11; 28, 21-46	30 ± 11; 24, 19-44	10-84
**Glucose (mg/dl)**	90 ± 14; 90, 68-107	94 ± 14; 95, 76-114	98 ± 11; 97, 84-116	89 ± 18; 89, 69-108	104 ± 26; 103, 74-136	95 ± 7; 95, 85-103	99 ± 5; 98, 93-105	103 ± 7; 100, 97-114	99 ± 5; 97, 95-107	65-130

Values are mean ± standard deviation, median and range. With the exception of lactate, groups with the same superscript are significantly different from each other within each row with respect to the time the sample was taken, P<0.01; however there were no differences between the skill groups. For lactate, groups with the same superscript are significantly different between skill groups and the time the sample was taken (i.e. pre and post exercise test, P<0.01). Pre = pre-exercise, Post = post-exercise, 4 hr post = 4 hours following exercise, BUN= blood urea nitrogen, ALT= alanine aminotransferase, ALP= alkaline phosphatase, mmol/L= millimoles per liter, mg/L= milligrams per deciliter, IU/L= international units per liter, mEq/L= milliequivalents per liter.

### Oxidant stress

Urinary creatinine excretion was 275.4 ±118.0 (median 262.9, range 41-593.5 mg/dL) pre-exercise, 205.7 ± 116.7 (median 213.1, range 29.9-446.8) mg/dL and at 4 hours post-exercise 223.8 ± 103.8 (median 222.1, range 36.7-418.2) mg/dL. While urinary creatinine was not affected by the exercise test, it was significantly less in the intermediate group of dogs compared to the other groups (P<0.01). Urinary excretion of ISO increased following agility exercise and remained elevated 4 hours after the exercise test [pre-exercise 14.50 ± 5.23 (median 14.62, range 2.48-20.70), post-exercise 20.31 ± 12.95 (median 16.20, range 9.46-60.70) and 4-hours post-exercise 19.00 ± 4.95 (median 18.44, range 12.05-26.75) pg/mL per mg/dL creatinine, P>0.05], however, this mild increase was not statistically significant.

### Urinary eicosanoid excretion

**Figure 1:**
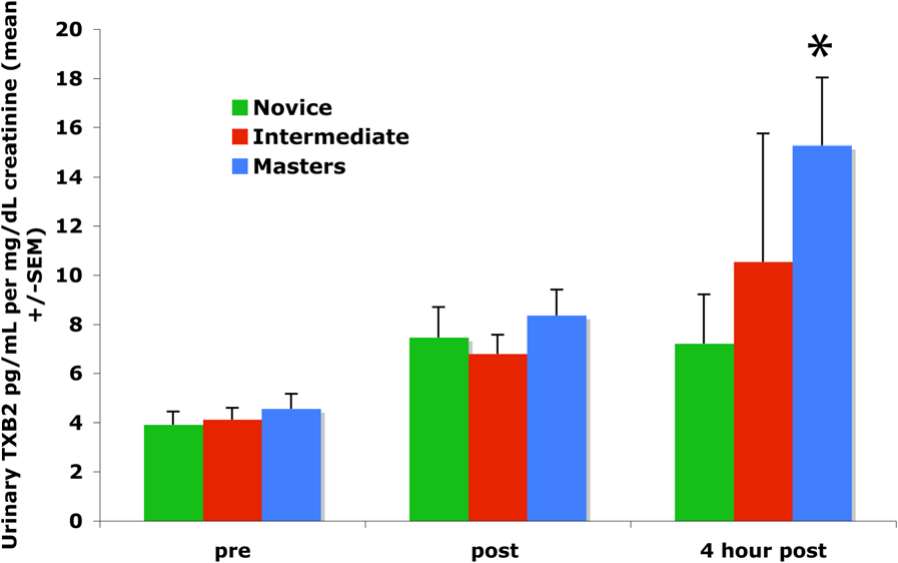
Urinary Thromboxane excretion by skill level in agility dogs, For all dogs compared to pre-exercise values TXB2 increased over time P=0.005, * = 4 hour post excretion elevated in masters dogs compared to pre- and immediate post-exercise, P=0.0081.

Urinary prostaglandin E_2_ metabolites (13,14-dihydro-15-keto-prostaglandin A_2_ and 13,14-dihydro-15-keto-prostaglandin E_2_) and 13,14-dihydro-15-keto prostaglandin F_2α_ were not affected by the agility exercise at any time point (Table, two-way ANOVA, P>0.05). Prostacyclin (6-keto prostaglandin F_1α_) excretion increased only in masters dogs compared to intermediate dogs at 4 hours following the exercise test (two-way ANOVA, Bonferroni post-test, P=0.04). Urinary TBX2 more than tripled by 4 hours post agility exercise (pre 4.56, post 11.76, 4 hour post 14.91 pg/ml/mg/dl creatinine, P=0.005, one-way ANOVA, GraphPad Prism 5.0a, La Jolla, CA) with a significant increase in masters dogs (pre 4.55 ± 0.62, post 8.35 ± 1.06, 4 hour post 15.27 ± 2.76, P=0.0097) but not novice (pre 4.50 ± 0.70, post 9.89 ± 2.67, 4 hour post 7.21 ± 2.01, P=0.19) or intermediate dogs (pre 6.68 ± 2.60, post 11.72 ± 4.97, 4 hour post 10.53 ± 5.22, P=0.08, one-way ANOVA), Figure 1. Urinary 11-TXB2 significantly increased over time (P=0.035) but did not differ between the skill groups: novice pre 19.07 ± 1.29, post 25.80 ± 1.43, 4 hour post 25.37 ± 1.82); intermediate dogs pre 5.49 ± 1.47, post 10.60 ± 0.74, 4 hour post 10.99 ± 0.66; masters pre 3.79 ± 1.69, post 15.15 ± 2.22, 4 hour post 13.65 ± 1.52 (P>0.05, two-way ANOVA).

## Discussion and conclusions

The skill levels of the dogs reported in this study was determined by the rules set out by the USDAA and may have seemed arbitrary. The groupings proved accurate in these dogs, however, since each successive skill group was significantly faster than the novice group and the group level below it. The labeling of these groups as novice, intermediate and masters could have been labeled instead by their time through the simulated exercise test, however, this would have been less applicable to dogs in actual agility competition.

Agility exercise, as performed by dogs in this study, is of sufficient intensity to induce mild oxidant stress and lipid peroxidation based upon the slightly increased urinary excretion of ISO following the exercise test. The intensity of muscle contraction associated with agility exercise increases production of reactive oxygen species due to increased oxygen consumption, resulting in elevated superoxide anion production in skeletal muscle mitochondria [19]. All dogs in this study had similar increases in ISO regardless of skill level and speed through the course, indicating that agility exercise routinely stimulates intense muscle contraction and results in mild lipid peroxidation. It is unlikely, given the results reported here, that the mild oxidant stress encountered in a single agility exercise session has a significant effect on physiologic function and performance. Previous reports have documented oxidative stress in dogs exercising for 20 minutes (hunting dogs) and many hours (sled dogs), however, this is the first report, to the authors’ knowledge, of the effect of very short duration, intense exercise on lipid peroxidation and oxidation in the dog [13,20,21]. Many agility dogs participate in more than one trial in a single day or weekend. In humans, intense exercise elevates urinary ISO excretion for 24 hours, and following a soccer game, humans experience oxidant stress and reduced performance for up to 72 hours [12,22]. Muscle fatigue due to oxidant stress may be a contributing factor to joint injuries, at least in humans [23,24]. Whether oxidant stress plays a role in injury to dogs during agility competition with repeated runs through a course in a single day remains unknown, however determining the level of oxidant stress that develops over time and the length of time before isoprostane production returns to pre-exercise levels in actual agility competition (rather than simulated as reported here) may be warranted based upon the results of this study and the high incidence of injury following participation in this activity [2]. Collection of urine at more precise time points following the agility exercise may have altered the results in our study by reducing variability between the subjects. Future research determining other markers of oxidant stress at exact time points prior to and following agility exercise is indicated by the results of this study.

A significant limitation of this study is the use of an EIA for determination of F_2_ isoprostanes in the urine of dogs. Analysis of the methods used to determine urinary F_2_ isoprostane excretion have found that EIA results do not correlate with gas chromatography negative ion chemical ionization-mass spectroscopy (GC/NICI-MS, considered the gold standard) in dogs [25]. The results of this study cannot be compared to other studies, however, mildly increased lipid peroxidation following the intense, short-duration exercise of agility competition occurred in the dogs reported here and was not affected by the speed or training of the dogs. Further research, preferably using GC/NICI-MS for urinary F_2_ isoprostane determination, is required to determine the extent of oxidant stress induced by this type of exercise in relation to long duration exercise in sled dogs and to disease states including lung injury [26], neurologic disease [16] and heart disease [27]. Future research is warranted to determine the effect of agility exercise on other parameters of oxidant stress including glutathione, glutathione-peroxidase, catalase, superoxide dismutase.

Another limitation of this study is the lack of data regarding antioxidant status in these dogs. Antioxidants are either supplements or endogenous mechanisms of consumption or removal of reactive oxygen species and include superoxide dismutase, catalase, glutathioperoxidase, glutathione reductase, vitamin E, vitamin C, and ubiquinone [28]. Hunting dogs exercised for 20 minutes to 4 hours duration decrease their biological antioxidant potential following the exercise but the return of this potential is within one hour [29]. Determination of antioxidant status may be warranted in future studies of dogs participating in agility exercise.

The plasma lactate increased markedly in the dogs following the exercise test. The changes in plasma lactate were inversely proportional to the speed through the course (and proportional to increasing skill level). Plasma lactate concentration at a specific speed or level of intensity is strongly correlated to physical fitness in horses as well as human athletes [30,31]. In the current study, all three skill groups had normal plasma lactate concentrations by 4 hours after exercise, indicating that recovery from lactic acidosis in agility dogs occurs quickly, similar to other athletic events, and before recovery from oxidative stress [32].

In Greyhounds following high intensity sprint exercise, plasma lactate increased to 29.3 meq/L or more, and similar to the dogs reported here, returns to baseline in less than 4 hours following the exercise [32-35]. During field trial competition, Labrador retrievers also increase plasma lactate concentrations similar to the masters level dogs in this study [36-38]. Increased plasma lactate was most likely the result of anaerobic metabolism in contracting muscles during the sprint exercise [32]. Studies of the Greyhound have indicated that this breed has higher concentrations of type II fast twitch muscle fibers [39]. These fibers have the greatest glycolytic/anaerobic capacity and therefore are capable of producing large amounts of lactate during high intensity exercise [40]. The relative amount of muscle fiber types present in the dogs in this study is unknown, however, a higher percentage of type II fibers may have resulted in greater increases in plasma lactate following the exercise test and significantly shorter times through the agility course. Elite sprinters in men and horses have higher proportions of type II fibers than their slower counterparts and plasma lactate has been shown to correlate with racing speed in thoroughbred horses and female cyclists [41-44].

Lactic acidosis and oxidant stress may be causes of muscle fatigue [45]. The mechanism by which reactive oxygen species cause muscle fatigue during intense exercise is not fully understood, however, supplementation with the antioxidant, N-acetylcysteine alleviates muscle fatigue in humans and animals [46-49]. Muscle fatigue due to oxidant stress may be a contributing factor to joint injuries, at least in humans [23,24]. Increasing lactate production by high intensity-contracting muscle will result in decreased ionized calcium release from the sarcolemma and contribute to muscle fatigue [50]. Muscle fatigue has been linked to increased bone strain in dogs experimentally, and may contribute to the development of stress fractures [51,52]. Further research is warranted to determine the muscle fiber types of elite agility competitors and whether, in competition, plasma lactate correlates with injury rates in these dogs.

Platelets increased immediately following the agility exercise but by 4 hours later had returned to values similar to pre-exercise; and at all time-points, values were within reference range for the laboratory (200-900 × 1000/μl). In humans, increased platelet count occurs with intense exercise and are thought to be mobilized from the spleen, bone marrow and lungs, but they may also increase due to hemoconcentration from respiratory water loss as a result of panting [53-55]. The small reduction in platelet counts following exercise may be due to plasma volume expansion that occurs during recovery or reduced catecholamine-induced platelet release [53,55].

Similar to previous reports in agility dogs, increases in red blood cell count, hemoglobin and hematocrit occurred in all groups immediately following exercise [3,4]. Increases in these variables without a rise in total plasma protein is consistent with splenic contraction with similar findings reported in Greyhounds after racing [56]. RBC count, HCT, and hemoglobin concentrations were decreased at 4-hours following the agility exercise test. In humans, during the hours following exercise, there is an increase in plasma volume termed “autohemodilution” resulting in decreased concentrations of RBC’s, HCT and hemoglobin [57-59]. This phenomenon may explain the decreased red blood cell parameters in the dogs reported here at 4-hours following exercise without alteration in protein or albumin concentrations as fluid from the interstitial space moves into the vascular space. To the authors’ knowledge, this is the first report indicating “autohemodilution” in dogs and further research is needed. Plasma albumin concentration increased immediately after exercise (P<0.01) but remained within reference range and returned to pre-exercise values within 4 hours, Table 1. No other alterations in hematological or biochemical parameters were found.

Increased TXB2 production without concomitant increase in prostaglandin E_2_ or PGF1α as reported here in agility dogs has also been reported in horses following treadmill exercise [60]. In endurance exercise, horses may also increase 6-keto-prostaglandin F_1α_ production, however this did not occur in the dogs of this study where the duration of exercise lasted less than 20 minutes [15]. The dogs with the greatest skill level (and fastest run time) had a significantly greater increase in TXB2 and this association has also been reported in horses [15]. High intensity exercise reportedly stimulates increased TXB2 production whereas submaximal exercise induces increases in PGF1α rather than TXB2 in humans [61]. Urinary 11-TXB2 has been determined to be a reflection of in vivo platelet activation in humans [62]. TXB2 in urine includes renal production of thromboxane A_2_ and may not reflect systemic circulation or platelet production of the unstable prostaglandin, thromboxane A_2_[63]. In this study both TXB2 and 11-TXB2 increased following exercise and remained increased 4 hours post exercise indicating systemic increases in thromboxane A2 occurred in the dogs. Interestingly, only TXB2 was significantly increased when skill levels were examined and not 11-TXB2 which may indicate greater renal production of thromboxane A2 only in the fastest dogs. Potential sources of TXB2 may include endothelial cells activated by shear stress or catecholamine activated platelets, however the source was not determined in this study. Future research to determine why improved performance is associated with increased TXB2 in the urine is warranted.

The dogs participating in the exercise test were not controlled for breed, age, sex, or nutritional status. Restriction of these variables was not enforced since a very wide variety of dogs participate in agility sports and many of them go on to injure themselves in this exercise; therefore, we sought to ascertain the degree of physiological alteration that occurs in a variety of agility dogs. Future research may limit the participants by breed etc. and investigate dogs during actual agility competition rather than a simulated test.

Agility competition in dogs has become a popular sport with a high incidence of injury [2]. Development of methods to reduce increases in urinary TXB2 and plasma lactate in elite canine athletes warrants investigation and determination of their effects on injury rates in dogs is a possible next step in this research. Possible methods to reduce these alterations might include improved maintenance of hydration with readily accessible water during agility exercise, adequate warm-up and cool-down exercise prior to participation in any agility exercise to improve musculoskeletal blood flow, and training methods that increase aerobic muscle capacity. Further understanding of the physiological responses to this type of intense, short-duration exercise may improve performance and reduce injury in dogs participating in this sport.

## Competing interests

The authors declare that they have no competing interests.

## Authors’ contributions

WIB carried out the study design, data collection, assay analysis, and drafted the manuscript. AMF designed the timing of the agility course, collected data, and participated in drafting the manuscript. BS performed the assays, standardized the analysis and participated in data interpretation. JJW participated in data collection and interpretation of the data. MEG performed the complete blood count and serum biochemistry analyses, interpreted the findings in these analyses, and participated in writing the conclusions and discussion involving these analyses. ECM participated in data collection, interpretation of the physical examination and cardiopulmonary data and helped to draft the manuscript. All authors read and approved the final manuscript.
